# 

**Published:** 1869-06

**Authors:** 


Hartford, Connecticut, June 10, 1869.

Eds. Dental Register: The ninth annual meeting of the American Dental Association will be held at Saratoga Springs, New York, commencing Tuesday, August 3, 1869, at 10 o'clock A. M.

The following form of certificate was adopted at the last meeting of the Association:

"This certifies that ——— was duly appointed a delegate to the American Dental Association on the — day of ——, 18—, by the Dental Society of ———, and that said ——— is a Dentist of good character and standing and at this time in regular practice."

No delegate will be admitted without he answers the requirements of this certificate, which he must bring with him, and present in person.

Accommodations will be secured for those giving early notice to Dr. J. G. Ambler, No. 25 West Twenty-third St., New York City.

There is reason to anticipate a profitable and enjoyable session, and it is hoped that representatives from every local society in the United States may be present.

James McManus,
Cor. Sec. American Dental Association.

### The Dental Register

### OF THE WEST,

*Issued Monthly, at $3 a Year in Advance.*

DEVOTED TO THE INTERESTS OF THE DENTAL PROFESSION.

EDITED BY J. TAFT AND G. WATT.

The volume commences in January and closes in December.

The Register being issued monthly is always fresh, therefore indispensable to the practitioner who desires to keep posted up with the new inventions and improvements which are daily developed. Neither expense nor effort will be spared to give value and variety to its contents. Articles will be illustrated with well executed engravings whenever they will add to the interest or assist in explanation.

Its extensive circulation and frequency of issue makes it one of the BEST DENTAL ADVERTISING MEDIUMS in the United States.

RATES OF ADVERTISING.

One page, 1 year, $50. Half page, 1 year, $30. Quarter page, or card, 1 year $20.

All advertising bills payable in advance, or at furthest after the issue of the first number containing the advertisement. All transient advertisements to be paid for in advance

☞ CONTRIBUTIONS SOLICITED.

Address, J. TAFT, or "DENTAL REGISTER," Cincinnati Ohio.

Business Communications to

J. TAFT, Publisher,
No. 117 West Fourth St.

